# Coherent organization of electronic correlations as a mechanism to enhance and stabilize high-*T*_C_ cuprate superconductivity

**DOI:** 10.1038/s41467-017-02422-2

**Published:** 2018-01-02

**Authors:** Haoxiang Li, Xiaoqing Zhou, Stephen Parham, Theodore J. Reber, Helmuth Berger, Gerald B. Arnold, Daniel S. Dessau

**Affiliations:** 1 Department of Physics, University of Colorado at Boulder, Boulder, CO 80309 USA; 2 Institute of Physics of Complex Matter, École Polytechnique Fédérale de Lausanne, CH-1015 Lausanne, Switzerland; 3 Center for Experiments on Quantum Materials, University of Colorado at Boulder, Boulder, CO 80309 USA

## Abstract

Strong diffusive or incoherent electronic correlations are the signature of the strange-metal normal state of the cuprate superconductors, with these correlations considered to be undressed or removed in the superconducting state. A critical question is if these correlations are responsible for the high-temperature superconductivity. Here, utilizing a development in the analysis of angle-resolved photoemission data, we show that the strange-metal correlations don’t simply disappear in the superconducting state, but are instead converted into a strongly renormalized coherent state, with stronger normal state correlations leading to stronger superconducting state renormalization. This conversion begins well above *T*_C_ at the onset of superconducting fluctuations and it greatly increases the number of states that can pair. Therefore, there is positive feedback––the superconductive pairing creates the conversion that in turn strengthens the pairing. Although such positive feedback should enhance a conventional pairing mechanism, it could potentially also sustain an electronic pairing mechanism.

## Abstract (web summary)

Whether the normal state electronic correlations in cuprates are responsible for superconductivity remains elusive. Here, Li et al. report that such correlations turn into a renormalized coherent state starting well above the superconducting transition, and it leads to a strengthened superconductive pairing.

## Introduction

Two main limits of electronic correlation effects exist in modern correlated electron metals—strongly coherent correlation effects and strongly diffusive incoherent correlations. Landau taught us the general principles for thinking of systems with coherent correlation effects, in which the quasiparticles (elementary excitations of the system) are the fundamental objects^1^. In these systems, strong interactions renormalize the quasiparticle energy-momentum dispersion relation, endowing the quasiparticles with large effective masses that in certain heavy Fermion systems may be 10 or more times larger than the non-interacting electron dispersion^2^. The huge majority of concepts in modern condensed matter physics build upon this quasiparticle foundation, including the Bardeen Cooper Schrieffer theory of superconductivity^3^ and the extensions such as the Eliashberg^4^ and Nambu–Gorkov theories^5,6^.

The second limit of correlated electron systems with diffusive or incoherent interactions is generally considered to be much more exotic and richer than the first class, though because we do not have Landau’s tools to visualize or understand the excitations, they are less understood. The non-superconducting normal or strange-metal state of the cuprate high-temperature superconductors is typically believed to belong to this second regime of correlated electron materials, and is generally considered to be among the most exotic of any electronic material. Almost no electronic property (magnetism, thermodynamics, optics, transport, etc.) of this state behaves like that in any other material, and it is believed by some that the strong interactions make the concept of a particle useless^7^. Efforts to understand these non-quasiparticle excitations are diverse, but often bring in the concept of fractionalized excitations^7,8^, utilize numerical methods to solve the Hubbard model^9,10^, and/or borrow strongly from string theory and quantum gravity^11–14^.

The superconducting state of the cuprates does not support these strange-metal excitations, instead, it has sharp quasiparticle-like excitations that somehow combine to form the Cooper pairs of the superconducting state. As the superconducting state is borne from the strange-metal normal state, it is natural to imagine that the diffusive correlations of the strange-metal state are somehow converted or re-organized to the interactions that are responsible for the superconducting pairing. Relatively few discussions along this line exist however, with most of them centering around the idea of an undressing of the strange-metal correlations^7,15–17^ to reveal what has sometimes been described as a hidden Fermi liquid state^18^.

With our development of the two-dimensional (2D) fitting technique for ARPES spectra, here we present the quantitative extraction of the fully causal complex electronic self-energies. Our approach can be considered an extended quasiparticle approach, which ultimately utilizes many of the concepts of quasiparticles but extends them via a broadening (imaginary part of the self-energy) that is sometimes (especially above *T*_C_) larger than the energy of the particles, i.e., these should not be considered true Landau quasiparticles. The extracted information of the electronic interactions directly confirms the general concept of the undressing of the strange-metal correlations and the unveiling of the hidden Fermi liquid, but it goes further by revealing a conversion or coherent reorganization of the diffusive strange-metal correlations into a coherent highly renormalized state at low temperature following by the enhancement of the number of states for pairing. We show how this can lead to a strong positive feedback effect that can stabilize and strengthen superconductive pairing, including favoring a strongly anisotropic (i.e., *d*-wave) superconducting gap. Such a mechanism with positive feedback not only allows for much stronger pairing than would be possible from a standard strong-coupling picture within the conventional electron–boson coupling scenario, it also has the potential to enable a fully electronic (non electron–boson mediated) mechanism of superconductive pairing.

## Results

### Experimental results and fits

**Fig. 1:**
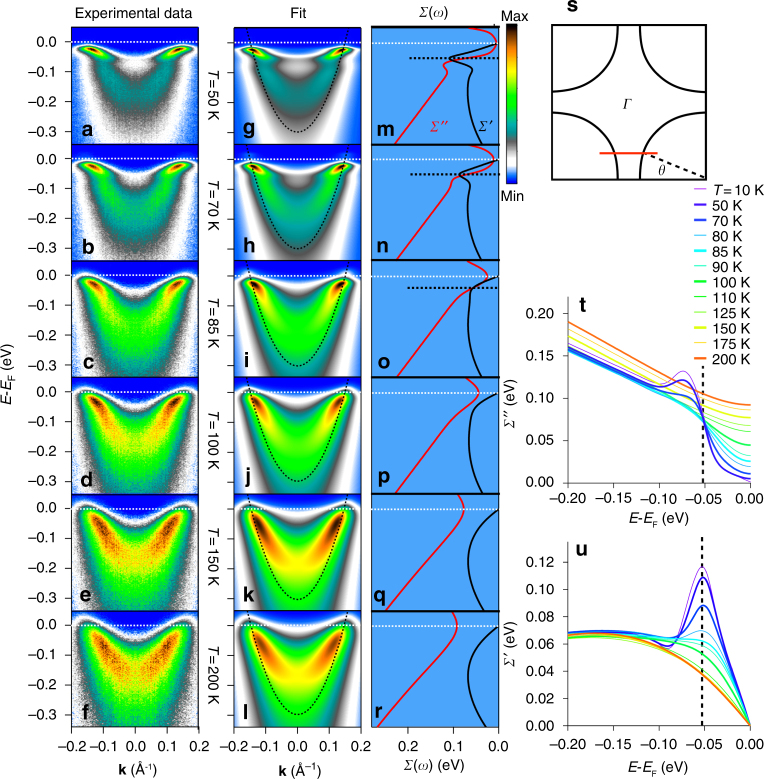
Temperature-dependent ARPES spectra and self-energies. **a**–**f** Measured spectra from a mid-zone cut (**s**) of the antibonding band at multiple temperatures. **g**–**l** 2D fits to the corresponding ARPES spectra, with the main unknown parameters the superconducting gaps, the electronic self-energies and the bare electron band, where the dotted curve is the bare-band dispersion from the fitting. **m**–**r** Correspondent self-energies extracted from the fit spectra on the left. **t** Imaginary part of the self-energy *Σ*″ (or scattering rate) vs. frequency and temperature obtained from the 2D fits. There is a strong reduction or gapping of the near *E*_F_ scattering rate as temperature is lowered into the paired state, along with a shift of this scattering to high energies beyond the dashed line. Along with this is a strong kink or mass enhancement in the real part of self-energy *Σ*′ (**u)**. These effects of *Σ*″ and *Σ*′ are responsible for the dramatic flattening and sharpening of the low-energy states of **a**–**f**, **g**–**l**. All data were taken with 9 eV photons, and the Fermi surface angle was *θ* = 22.5 degree

**Fig. 2:**
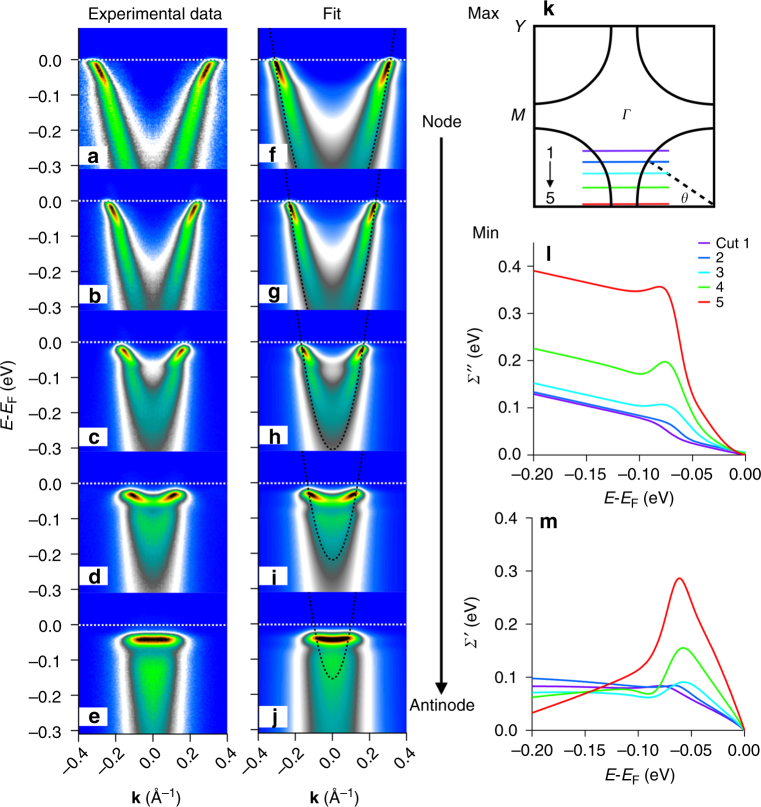
Dramatic growth of self-energies towards the antinodal regime. **a**–**e** Measured *T* = 15 K superconducting state ARPES data along cuts 1–5, as shown in **k**. In **e**, the states near *E*_F_ are almost dispersionless, i.e., extremely massive. **f**–**j** Corresponding 2D fits of the spectra, where the dotted curve is the bare-band dispersion from the fitting. **l** Extracted imaginary part of the self-energy *Σ*″ at the five cuts in k-space. **m** Extracted real self-energy *Σ*′ at the 5 cuts in k-space. The huge mass enhancement of the low-energy antinodal states of cut 5 is captured by the large magnitudes of *Σ*′ and *Σ*″ for cut 5. All data were taken with 24 eV photons, and the sample was the same as used for Fig. 1

Figures 1 and 2 show raw data sets and matching 2D fits from a lightly under-doped *T*_C_ = 85 K (Bi,Pb)_2_Sr_2_CaCu_2_O_8_ superconductor. The Pb-doping removes the 5 × 1 superstructure that can add contamination to the antinodal portions of the spectra, and experimental photon energies have been judiciously chosen so as to select only the antibonding band from the bilayer-split^19,20^ band structure (see Methods). The data were taken over a rather large energy range so as to capture the large energy scale features as well as the low-energy scale features such as the superconducting gaps. The data from Fig. 1 were taken near the middle of the zone (partway between the node and antinode, cut shown in Fig. 1s) and show the evolution of the various features as a function of temperature from deep in the superconducting state into the normal state. Figure 2 was taken in the superconducting state and shows how these features evolve as we move from near the node towards the antinode.

We use the conventional Nambu–Gorkov formalism for superconductivity^5,6^ for theoretically describing these spectra:

1 $$G({\bf{k}},\omega ) = \frac{1}{{\left[ {\omega - {\it{\Sigma }}} \right]^2 - \xi _{\bf{k}}^2 - \phi ^2}}\left( {\begin{array}{*{20}{c}} {\omega - {\it{\Sigma }} + \xi _{\bf{k}}} & { - \phi } \\ { - \phi } & {\omega - {\it{\Sigma }} - \xi _{\bf{k}}} \end{array}} \right)$$

where *G*(**k,** *ω*) is the electron propagator or Green’s function with the ARPES spectral function (intensity) *A*(**k**, *ω*) = −Im*G*_11_(**k**, *ω*)/π. In this compact notation (see Supplementary Note 2) *ξ*_**k**_ is the bare-band dispersion, *Σ* is the energy-dependent self-energy term that modifies the bare energies (real part of the self-energy *Σ*′ and widths, imaginary part of the self-energy *Σ*″), and *ϕ* is the pairing order parameter (with *ϕ* = *Z*(*ω*) × *Δ*, where $Z(\omega ) = 1 - \frac{\it {{\Sigma ^{\prime}}(\omega )}}{\omega }$ and *Δ* is the superconducting gap, more details contained in Supplementary Notes 1–9). In essence, we are using a conventional quasiparticle-like approach for both the normal and superconducting states, with the exception that there is a complicated and large self-energy term *Σ* that captures the interactions (and the departure from true Landau quasiparticle physics). That both the superconducting state and normal state spectra can be so well described within this semi-conventional approach is in itself a surprising finding: the generally broad structures and large backgrounds observed in ARPES spectra of cuprates have often been described as being so far outside the realm of conventional physics that such semi-conventional quasiparticle-like approaches were considered untenable^7^.

The key new information obtained from our study is that of the self-energies *Σ* = *Σ′* + i*Σ*″ (directly related to the electronic interactions), which we find vary dramatically as a function of energy, temperature, and from node to antinode, as shown in Figs. 1t, u, and 2l, m. Supplementary Notes 1–9, and Supplementary Figs. 2–7 contain more details of the fitting technologies and constraints that have enabled the extension of ARPES into a quantitatively accurate spectroscopy of the self-energies, while Supplementary Note 6 compares the fitting quality with that obtained using standard one-dimensional (1D) energy distribution curve (EDC) and momentum distribution curve (MDC) based techniques.

We initially focus on the spectral widths or scattering rates (*Σ*″) as shown in Fig. 1t, beginning with the high temperature (*T* ≥ 125 K) normal state data, which is smoothly varying as a function of energy, with a linear-like dependence on energy at high energies and a quadratic-like dependence at lower energies. The data also show that the overall value of the scattering rate at all energies in this high-temperature regime increases smoothly with temperature. Both of these are the expected behavior of the strange metal^21^ or Marginal Fermi Liquid state^22^, or more precisely the Power Law Liquid state^23^. Beyond just the functional form of *Σ*″ we also get information about the absolute value of the scattering rates, which are in general very high—ranging from 100 meV at *E*_F_ to about 190 at 200 meV binding energy for the 200 K spectrum, as shown in Fig. 1t. These large scattering rates are the reason for the overall broad spectra shown in Fig. 1e, f, k, l, with the broadening somewhat larger at deeper energies as described by the curves of Fig. 1t. These large normal state scattering rates are a critical aspect of the strongly correlated state of the cuprates, which we will come back to later.

As the sample is cooled below ~125 K we begin to see subtle and then more dramatic changes in the spectrum of *Σ*″, with a slow and then rapid decrease of *Σ*″ in the low-energy (<60 meV) portion of the spectra. With the decrease in *Σ*″ at low energies, the low-energy peaks in the spectral function become noticeably sharper, as seen most clearly in panels a and g, qualitatively consistent with previous ARPES results^24–28^. The sharp energy step in the undressed *Σ*″ brings in dramatic changes in *Σ*′. In particular, the low temperature step increases in *Σ*″ centered at approximately 55 meV leads to a strong peak in *Σ*′ at that same energy due to the Kramers–Kronig relations that derive from causality. This effect is absent at the highest temperature and strongest at the lowest temperatures. *Σ*′ renormalizes the band energies, pushing the bare-band energies *ξ*_*k*_ toward *E*_F_ by the amount of *Σ*′, leading to two key consequences: a dispersion kink appears at the energy where *Σ*′ is maximal (~55 meV) and the dispersion of the low-energy bands are flattened/made more massive, by an amount equal to the renormalization factor $Z = 1 - \left. {\frac{{{\it \partial \Sigma ^{\prime}}}}{{\partial \omega }}} \right|_{\omega = E_{\mathrm{F}}} \sim 3$ for the low temperature data of Fig. 1, i.e., panels a and g. The same energy scale appears as the step in *Σ*″, peak in *Σ*′, and kink in *A*(**k**, *ω*) and is ~55 meV, consistent with previous results from the antinodal regime^25–28^

Figure 2 shows how the low temperature self-energies vary across the Brillouin zone, moving from the near-nodal regime (top) to the antinodal regime (bottom). In addition to the superconducting gap in the spectral function growing as we move to the antinode we see that the quasiparticle dispersion becomes flatter and flatter from node to antinode, with a correspondingly stronger dispersion kink or renormalization effect. This evolution of the renormalization effect is seen in both *Σ*″ (Fig. 2l) and *Σ*′ (Fig. 2m), as the self-energies are in general larger and more dramatically varying at the antinode than at the node. In particular, *Σ*″ in the high energy portion of the antinodal spectrum is extremely large with a magnitude ~350 meV, compared with the still-large magnitude of ~120 meV for the near-nodal spectrum. Because the very low-energy/low temperature portion of *Σ*″ has an extremely small value of ~2 meV, this means that the antinodal states have a much stronger step decrease in the scattering rate *Σ*″ with energy and a correspondingly stronger peak in *Σ*′, giving rise to a huge kink effect and almost dispersionless (extremely massive) states near *E*_F_ (Fig. 2e, j). Therefore, the larger the *Σ*″ is, the larger is the kink in *Σ*′.

### Conversion of self-energy

**Fig. 3:**
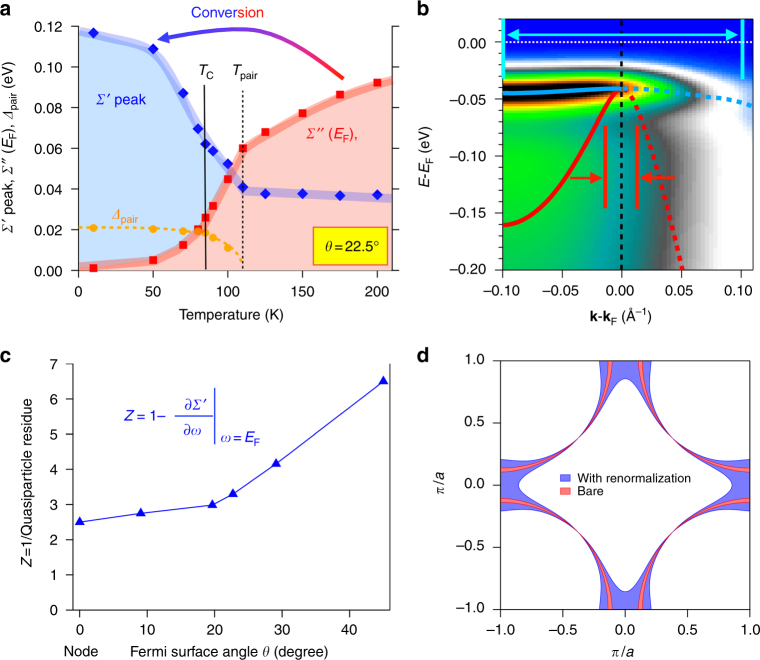
More details of the effects of the self-energies. **a** Temperature dependence of gaps and self-energies of the *θ = *22.5° mid-zone cut of Fig. 1. Parameters shown are *Σ*′ peak (blue diamonds −*Σ*′ value along the dashed line in Fig. 1u), *Σ*″(*E*_F_) (red squares—zero frequency portion of Fig. 1t), and superconducting gaps (orange circles—extracted from 2D fits). Upon cooling from high temperature, the main evolution of the parameters begins at *T*_pair_ and not at *T*_C_. **b** Reproduction of the right half of the spectrum of Fig. 2j (antinodal states, cut 5, *T* = 15 K), with the renormalized quasiparticle dispersion (blue curve), and the extracted bare-band dispersion with no self-energies (red curve) overlaid on the spectrum. The superconducting gap is 40 meV. The red (bare) and blue (with renormalization) arrows indicate the effective range of **k**-states contributing to particle-hole mixing and pairing (see Supplementary Note 10). This effective **k** range is significantly larger for the renormalized band (blue) than for the bare band (red). **c** Quasiparticle renormalization factor *Z* (blue triangles) in the superconducting state as a function of Fermi surface angle, reaching the very large value of 6.5 at the antinode. **d** The effective **k** range for pairing in the Brillouin zone, where the effective **k** range with renormalization covers 11% Brillouin zone, the bare one only covers 2% Brillouin zone

In Fig. 3a, we plot the temperature evolution of the renormalization effects at the mid-zone cut of 22.5 degrees (raw data in Fig. 1) as well as the temperature evolution of the superconducting energy gap from that same data set. *Σ*″(*E*_F_) (red squares of Fig. 3a) is seen to decrease gradually with decreasing temperature (as expected for a Marginal Fermi Liquid^22^ or Power Law Liquid metal^23^), but then take a steep dive at the onset of the superconductive pairing *T*_pair_, as also determined by the temperature at which the pairing gap *Δ* reaches zero (orange circles of Fig. 3a). Concomitant with the drop in *Σ*″ is a rapid rise in *Σ*′ (blue diamonds of Fig. 3a), i.e., the large imaginary part of the self-energy is converted into a large real part of self-energy (and hence large renormalization factor *Z*) as the temperature is lowered and the pairing fluctuations begin.

Figure 3b shows the fit of the spectrum at the antinode at 15 K (same as the right half of Fig. 2j) together with the quasiparticle locus (renormalized dispersion—blue) and the gapped bare band (red). The renormalization at the antinode is so huge that the quasiparticle dispersion at the gap edge is almost completely flat. This effect is characterized by the renormalization factor *Z* (equivalent to the inverse of the quasiparticle residue, more details contained in Supplementary Note 11), which is 6.5 for the present case at the antinode, with a value slightly <3 near the node (blue triangles, Fig. 3c). In a non-gapped metal this renormalization parameter would also be the mass enhancement m*/*m* (ratio of second derivatives of the band dispersion). Such a huge renormalization factor and mass enhancement is very unconventional, partly because obtaining such a strong coupling via an Eliashberg electron–phonon interaction would typically cause a different instability (such as a charge density wave) that will compete with the superconductivity. Our data and discussion below argue that in this case the superconductivity is intimately related to (and self-consistently drives) the huge coupling, so this is very different from the conventional theory.

### Enhancement of the number of states for pairing

A critical aspect of the huge band renormalization/mass enhancement is that it brings many states that were originally far from *E*_F_ up toward the Fermi level. If these new states are brought within an energy scale comparable to the pairing energy (within $\sqrt 2 {\mathrm{\it \Delta }}$ of *E*_F_—see Supplementary Note 10), then these new states will strongly participate in the superconductive pairing. This effect is illustrated in Fig. 3b, where there is an approximately 7-fold increase in the number of k-states contributing to the particle-hole mixing of superconducting pairing. As for the entire Brillouin zone (Fig. 3d), we find that this effect brings a 5-fold increase in the number of paired **k**-states (blue) compared with the situation without the renormalization effect.

**Fig. 4:**
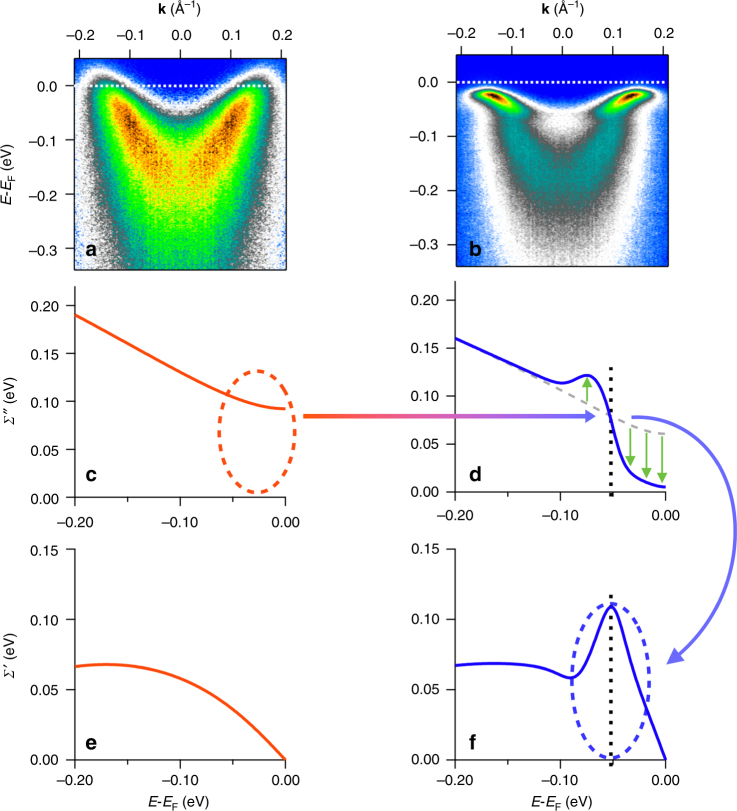
Conversion of electronic correlations. This figure summarizes the temperature evolution of the electron spectral weight *A*(**k**, *ω*) **a**, **b** and the electronic self-energy *Σ*(**k**, *ω*) **c**–**f**. **c** highlights the large low-energy *Σ*″ (incoherent scattering) that is present in the normal state, as indicated by the dashed oval. **d** shows that as the temperature is lowered into the superconducting state this low-energy incoherent scattering is undressed or gapped, i.e., the low-energy portion of *Σ*″ tends toward zero below the energy of the dashed line, along with a slight increase in *Σ*″ at higher energy (partial weight transfer). Among other things this creates the well-defined quasiparticle states in *A*(**k**, *ω*) at low energy of **b**. Causality requires that associated with this change in *Σ*″ is a change in *Σ*′, as observed in **f** and highlighted by the blue dashed oval. This shows up as the strong kink effect/mass renormalization in **b**. Hence, the large diffusive scattering above *T*_C_ is converted to a strong kink effect and mass renormalization—an effect that is much larger for the antinode than the node because the normal state diffusive scattering is much larger for the antinode than the for the node

Figure 4 summarizes the temperature evolution of the electron spectral function and the self-energy conversion, i.e., how the correlation effects are converted from giving a strongly diffusive (large *Σ*″) low mass state at high temperatures to a coherent (low *Σ*″) mass-enhanced state (*Z* or *Σ*′ effect) in the superconducting state. In particular, at the first formation of a gap in the spectral function *A*(**k**, *ω*) at around *T*_pair_, we start to get a gapping of the low-energy portion of *Σ*″, as illustrated in panel d. The newly created steep step increases in *Σ*″ automatically (through the Kramers–Kronig relations) leads to a strong peak in *Σ*′ (Fig. 4f). The stronger is the step in *Σ*″, the stronger is the peak in *Σ*′ and the larger the dispersion kink and mass renormalization, as quantified by the magnitude of *Σ*′ and *Z*. Within this picture the huge (6.5-fold at the antinode) renormalization that we observe in the superconducting state is therefore directly attributable to the anomalously large diffusive scattering rate in the normal state. This large mass enhancement is beyond the simple concepts of undressing of the normal state correlations as discussed in the previous works^7,15^, as in those ideas the normal state correlations were removed, not converted. The conversion effect described here also has similarities to the Kondo effect observed in heavy Fermion materials, in which high-temperature incoherent correlations give rise to a highly massive coherent state at low temperature^2^.

## Discussion

**Fig. 5:**
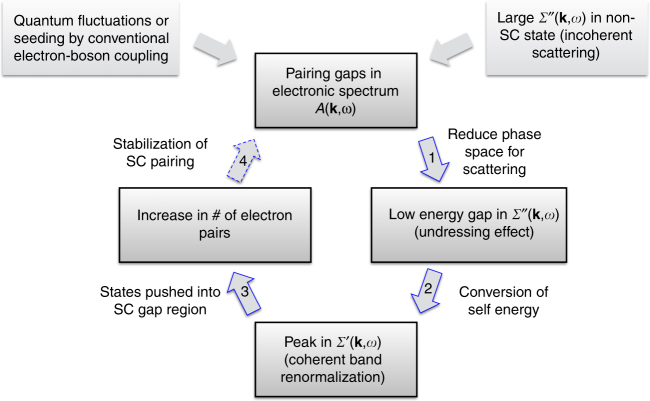
Proposed positive feedback effect between *A*(**k**, *ω*) and *Σ*(**k**, *ω*). (1) The onset of pairing fluctuations (gapping) in the electronic spectrum *A*(**k**, *ω*) will start to open a gap in the scattering spectrum *Σ*″ because of the removal of the low-energy electronic states. (2) Then as shown in Fig. 4 the gapping and weight transfer in *Σ*″ leads to strong peaks in *Σ*′, which give a strong mass enhancement in *A*(**k**, *ω*). (3) This low-energy band renormalization brings an increase in the number of low-energy **k**-states available to participate in the pairing (Fig. 3b, d), i.e., the effect of the pairing on *A*(**k**, *ω*) is enhanced by the changes in *Σ*(**k**, *ω*). (4) This enhancement in the pairing can feed back to the original pairing fluctuations of step 1, helping to strengthen and stabilize them. This mechanism is possible because of, or enhanced by, the huge incoherent scattering rate *Σ*″ present in the strange-metal incoherent normal state

Our observations point toward the possibility for a positive feedback on the pairing process that can markedly enhance and stabilize superconductive pairing. The idea is illustrated in Fig. 5, and is as follows: (1) Any onset of pairing fluctuations in the electronic spectrum *A*(**k**, *ω*) will start to open a gap in *A*(**k**, *ω*), which due to the removal of low-energy electrons from which to scatter, will reduce the phase space for electron-electron scattering. This reduced phase space for low-energy electron–electron scattering implies the opening of a gap in the low-energy portion of *Σ*″. (2) Because of causality and the Kramers–Kronig relations, the gapping in *Σ*″ leads to strong peaks in *Σ*′ which give a strong coherent band renormalization effect in *A*(**k**, *ω*) (Fig. 1) that is strongest for the antinodal states (Fig. 2). (3) This low-energy coherent band renormalization brings an increase in the number of low-energy **k**-states available to participate in the pairing (Fig. 3b, d), i.e., the effect of the pairing on *A*(**k**, *ω*) is enhanced by the changes in *Σ*(**k**, *ω*). (4) As the gap in *A*(**k**, *ω*) enhances the changes in *Σ*(**k**, *ω*), and the changes in *Σ*(**k**, *ω*) enhance the changes in *A*(**k**, *ω*), we propose that a positive feedback loop is created that should strengthen and stabilize the superconductive pairing. This feedback effect should favor an anisotropic (e.g., *d*-wave) pairing channel as the self-energy effects are much greater at the antinode than near the node (Figs. 2l, m and 3c), and the effect can in general be very strong because of the very large overall strength of *Σ*″ in these materials.

Among other things, our work also shows the next step in the technical analysis of ARPES data, effectively turning ARPES into a self-energy spectroscopy. Over the past years, ARPES has made great progress at revealing certain aspects of the electronic interactions (characterized by the self-energies) of the cuprates. In particular, ARPES has discovered and provided key details of the unusual incoherent scattering or peak broadening^23,25,29^ that is consistent with the strange-metal transport^21^ or Marginal Fermi Liquid state^22^, or more precisely the Power Law Liquid state^23^. In contrast, using the standard language of peak tracking of quasiparticles, ARPES has described dispersion kinks or mass enhancements in the superconducting state in the nodal^24,30–32^ and antinodal regime^25–28,^ which have been generally interpreted as indicating rather conventional electron-–boson coupling, of the type for example that may act to pair electrons in a conventional non strongly-coupled electronic material. However, prior to the present work, ARPES analysis only focus on one-dimensional energy cuts (EDCs) or one-dimensional momentum cuts (MDCs)^17,23,33^, but not both together—an issue that is not important when the peaks are sharp and the scattering rates are low like in a Fermi Liquid. In strongly correlated superconductors like cuprates, the complications of the peak broadening, the renormalization effect, and the spectral gap make a quantitative extraction of the band dispersions and self-energies from ARPES possible only in certain special cases. In particular, along the nodal direction where there is a linearly dispersing band with relatively high velocity, the self-energies can be extracted through MDC analysis^23,30–32^, but this method fails for mid-zone or antinodal cuts, in which more complicated structures come in with the spectral gap and a strong kink feature below *T*_pair_^25–28^. Whereas near the antinode, the sharp quasiparticle peak below *T*_C_ allows the relatively flat band dispersion to be extracted by EDC analysis, the EDC method fails when the gap starts to disappear near or above *T*_C_^26–28^. Thus, the MDC and EDC methods can only provide qualitative result in certain cases, and in the general case they may give very different results from each other^34^. Therefore, previous studies have only been able to focus on certain parts of the Brillouin zone or certain temperature ranges (below or above *T*_C_) and these studies only extracted qualitative feature from part of the self-energy (either the real or imaginary part within a certain energy range)^24–28,30–32^.

Our work provides an analysis tool of a full 2D fitting technique, treating both EDCs and MDCs simultaneously and on an equal footing, while also drastically reducing the number of free fitting parameters (see Supplementary Notes 1 and 2). Another important advance of this technique is that the self-energy *Σ*(**k**, *ω*) has been constrained to be automatically Kramers–Kronig (KK) consistent, i.e., the obtained self-energies explicitly obey causality, whereas the 1D MDC and EDC methods have no restrictions on causality (Supplementary Notes 2–4). The self-energies extracted from spectra covering a wide temperature range and the full Brillouin zone provide a more comprehensive picture of the electronic interactions (Figs. 1, 2, and 3) that was not achievable in the previous studies with simple one-dimensional MDC or EDC methods. The extracted self-energies shown in Figs. 1, 2, and 3 not only quantitatively confirm the undressing behavior of *Σ*″ and the kink feature as the peak of *Σ*′ from previous studies, but more importantly they provide the direct observation of the conversion of the self-energy as shown in Fig. 1t, u, and Fig. 3a. Moreover, we present the evolution of the electron correlation effects from different parts of the Brillouin zone, with these strong renormalization effects providing a strong enhancement to the pairing state as shown in Fig. 3d, giving evidence of a positive feedback loop as described earlier in this discussion and illustrated in Fig. 5.

The strength of our observed coupling is unprecedented in superconductors. The typical theory for dealing with strong coupling is Eliashberg theory. The standard Eliashberg equations have been derived for conventional superconductors where the Cooper pairing is mediated by an electron–boson interaction. A key starting point for the Eliashberg equations is Migdal’s theorem^35^, in which the energies or velocities of the electrons are much greater than those of the bosons, and thus the vertex corrections can be ignored and the coupling strength is a constant. Thus, the standard Eliashberg theory is adiabatic and semi-perturbative, i.e., it does include higher order interaction diagrams, but it neglects higher order diagrams from the vertex correction. On the other hand, the maximum electron–phonon interaction strength for driving superconductivity is limited owing to the stability of the lattice. Therefore, a renormalization factor (*Z*) up to 6.5 in a delocalized superconductor is unexpected and likely impossible in a standard Eliashberg electron–phonon theory.

Our data also question the concept of adiabiticity, i.e., the assumption that the electrons are much faster or higher energy scale than the bosons. Our data show that the electron energy at the antinode is ~40 meV (Fig. 3b), which is nominally the same energy as the bosonic degrees of freedom most discussed as coupling to the electrons (neutron resonance mode ~40 meV^36^, B_1g_ phonon ~40 meV^28^, LO phonon ~55 meV^30^), and also lower energy than our kink energy of ~55 meV (Fig. 1t, u).

In general, a positive feedback or bootstrapping mechanism such as we describe has the potential to strengthen and stabilize any type of superconductive pairing mechanism, regardless of the initial mechanism of pairing (electron–phonon, electron-spin resonance, spinon pairing/RVB physics, etc.). More interestingly, we note that this mechanism also gives a potential route toward a purely electronic pairing mechanism, where the initial pairing gap in *A*(**k**, *ω*) could be seeded by quantum fluctuations alone. Regardless of the specific mechanism for the pairing, the strength of the positive feedback effect depends upon the strength of the incoherent normal state scattering, and presumably also the details of this incoherent scattering. Of course, understanding the details of this diffusive normal state self-energy at high temperatures also has remained elusive, capturing the attention of physicists from a great range of disciplines^9,11,12^. That the interactions (*Σ*′ and *Σ*″) that drive this strange-metal state are essentially fully gapped when the low-energy electrons in *A*(**k**, *ω*) are gapped, is in itself an important clue about the origin of the strange-metal state, which should therefore be largely or fully electronic in origin. Even more, seeing how this strong diffusive scattering can be converted to strong coherent effects that can enhance and stabilize superconductivity opens a potential new path for engineering such effects into other materials, possibly with higher transition temperatures.

## Methods

### ARPES experiments

The ARPES experiments were performed on single crystals of lightly under-doped (*T*_C_ = 85 K) Pb-Bi2212, with the Pb-doping serving to minimize the effect of the superstructure modulations on the electronic structure^37,38^. ARPES measurements were taken at beamline 5–4 Stanford Synchrotron Radiation Lightsource, and at beamline 9A at the Hiroshima Synchrotron Radiation Source. The samples were cleaved and measured in an ultra-high vacuum chamber with the pressure maintained below 3 × 10^−11^ Torr. We used 9 eV photons for the temperature-dependent set of data in Fig. 1 and 24 eV photons to cover the full Brillouin zone (data in Fig. 2). These two photon energies are selected to minimize the matrix element of the bonding band and enhance the antibonding band. The energy resolution was 5.5 meV with 9 eV photons and 10.5 meV with 24 eV photons.

### Data analysis

The count rate nonlinearity of the electron detector is corrected^39^. All the spectra in the paper are symmetrized along *k* = 0. A weak energy-dependent background, presumably from elastically scattered electrons, is subtracted from each spectrum before fitting. This background is determined by measuring the counts at the edge of the spectrum as shown in Supplementary Fig. 1. The details of the 2D fitting method are discussed in the Supplementary Notes 2–4.

### Data availability

The data that support the plots within this paper and other finding of this study are available from the corresponding author on reasonable request.

## Electronic supplementary material

Supplementary Information
