# Supplementary material for: Coherent organization of electronic correlations as a mechanism to enhance and stabilize high-TC cuprate superconductivity
Source: Nat Commun. 2018 Jan 2;9:26. doi: 10.1038/s41467-017-02422-2 (PMC5750216; doi:10.1038/s41467-017-02422-2)
Supplement: Supplementary file 1 — Supplementary Information [file 41467_2017_2422_MOESM1_ESM.pdf]

Supplementary Note 1. 1D vs 2D fitting

For many years, the major technique of extracting self-energy effect from ARPES spectra were one-dimensional (1D) analysis methods using either energy distribution curves (EDCs) or momentum distribution curves (MDCs). However, with the presence of the strong renormalization effects and the superconducting gap, there are substantial discrepancies between the EDC and MDC methods[1]. In addition, with simply 1D analysis methods of MDCs or EDCs, it is difficult to obtain the full information in the spectral function, especially the energy-dependent self-energy of which the real and imaginary parts adhere to causality. Moreover, slight warpage, doping heterogeneity[2], or any other surface conditions of the sample may lead to undesirable broadening in the spectra, which add in more complexity for extracting the intrinsic broadening effects through 1D methods. These problems can be overcome with the two-dimensional (2D) fitting of spectral function convolved with extrinsic energy and momentum broadening and forcing the real and imaginary parts of self-energy to have full causality. Our 2D fitting method also dramatically reduces the ratio of fitting parameters to data points compared with the 1D method, which we will discuss later.

Supplementary Note 2. Nambu-Gorkov form of gap and self-energy

The entire ARPES cut as shown in Fig. 1 and Fig. 2 is fitted to a spectral function multiplied by a Fermi function and broadened by experimental resolution, which can be written as:

$$I_{\mathrm{ARPES}} = I_0[A(\mathbf{k},\omega)\times F(\omega,T)] \otimes R(\omega,\mathbf{k}) \quad (1)$$

where I_0 is the prefactor of spectral intensity, $A(\mathbf{k},\omega) = -\mathrm{Im}G(\mathbf{k},\omega)/\pi$ is the spectral function (imaginary part of the Green's function), $F(\omega,T)$ is the Fermi function and $R(\omega,\mathbf{k})$ is broadening function in both energy and momentum. We use the conventional Nambu-Gorkov formalism for

superconductivity[3,4] for theoretically describing these spectra. The 2×2 Green's function matrix is:

$$G(k,\omega) = \frac{1}{[\omega-\Sigma(\omega)]^2-\xi_{\mathbf{k}}{}^2-\phi^2}\begin{pmatrix} \omega-\Sigma(\omega)+\xi_{\mathbf{k}} & -\phi \\ -\phi & \omega-\Sigma(\omega)-\xi_{\mathbf{k}} \end{pmatrix} \quad (2)$$

The G_{11} term describes the electron removal portion, which is:

$$G_{11}(k,\omega) = \frac{\omega-\Sigma(\omega)+\xi_{\mathbf{k}}}{(\omega-\Sigma(\omega))^2-\xi_{\mathbf{k}}^2-\phi(\omega)^2} \quad (3)$$

where $\Sigma(\omega)$ is the complex self-energy, $\xi_{\mathbf{k}}$ is the bare band, ϕ is defined as $\phi = Z(\omega)\times\Delta_{\mathbf{k}}$ with $Z(\omega) = 1 - \frac{\Sigma'(\omega)}{\omega}$, and $\Delta_{\mathbf{k}}$ is the superconducting pairing gap. As all the two dimensional ARPES spectra in this paper are taken along the (π, 0) – (π,π) direction, a parabolic function with only two parameters (band bottom and $\mathbf{k}_F$ position) is used as the bare band instead of a more complicated tight binding model. We construct the phenomenological imaginary self-energy Σ'' for each spectrum with minimal parameters and use the Kramers-Kronig relation, which is $\Sigma'(\omega) = \frac{1}{\pi}\int \frac{\Sigma''(\omega')}{\omega'-\omega}d\omega'$, to obtain the real part. We chose the integration range to be ±5 eV, such that a larger range does not change the integration result. While we allow for particle-hole asymmetry for the electronic structure (i.e. parabolic band dispersions), we have constrained the self-energies to be particle-hole symmetric. We found that eight parameters in total are sufficient for constructing the self-energy for any one cut. We will discuss the details of constructing the self-energy later. Together with the extrinsic broadening terms (one for energy resolution and one for momentum broadening accounting for surface condition and sample heterogeneity), superconducting gap (real constant, but also checked for a complex energy-dependent option – Supplementary Note 7), bare band dispersion (two parameters), and the prefactor for the spectral function, there are only 14 fitting parameters in total for the two dimensional ARPES spectra below T_C. Each two-dimensional ARPES spectrum in this paper has over 10^5 data points. The

ratio of fitting parameters to data points is therefore ~1/6000. In a typical EDC or MDC analysis, a functional form of about 5 parameters is used to fit each EDC or MDC that contains about 300 to 400 data points (parameters to data points ~1/60). Therefore, the ratio of parameters to data points in our 2D fitting method is two orders of magnitudes smaller than the 1D analysis technique. Some previous studies have simulated ARPES spectra with a theory based or phenomenological self-energy to achieve reasonably good simulation result that resemble the experimental data taken from cuprates well below the superconducting transition[5,6]. Our analysis technique is the first fitting method that applies to the ARPES spectra with temperature ranging from well below the superconducting transition to well above it and with spectra taken from the node to the antinode.

Supplementary Note 3. Impact of the form of the self-energy

To show the construction of the self-energy, Supplementary Fig.2a-f demonstrates how the gapped imaginary self-energy and the kink in the real part of the self-energy affect the ARPES spectra. All spectra in Supplementary Fig. 2 are simulated with the same superconducting gap and bare dispersion with the parameters extracted from fitting to spectra in Fig. 2 panel d. Supplementary Fig. 2a shows a simulated spectrum with self-energy of the Marginal Fermi liquid form[7] (more specifically the Power Law Liquid form[8] as shown in Supplementary Fig.2b, which can be written as:

$$\Sigma''(\omega) = \lambda\sqrt{\omega^2 + (\pi k_{\mathrm{B}} T)^2} + \Gamma_0 \quad (4)$$

where λ is set to 0.5, Γ_0 is 80 meV, and T is 15 K. The real part of the self-energy is obtained from the Kramers-Kronig relation. Compared with the ARPES data (Fig. 2d in the main paper), the example spectrum of Supplementary Fig. 2a shows little feature from the superconducting gap (even though the gap has been input to the simulation) and it lacks the sharp feature near E_F

as well as a strong band renormalization. Then in Supplementary Fig. 2c, we replaced Σ' with one equivalent to the one extracted from our 2D fitting method (Supplementary Fig. 2d). Because we haven't yet adjusted Σ'', Σ'' and Σ' no longer adhere to the Kramers-Kronig relation. Although the spectrum shows a strong band renormalization from the Σ', there is still no sharp spectral peak and little feature from the superconducting gap due to the large Σ'' that broadens the quasiparticle coherence peak and heavily fills in the superconducting gap. In the next step (Supplementary Fig. 2e), we put in the full self-energy that is extracted from our 2D fitting method, which especially includes a strong drop in the scattering rate Σ'' at low frequencies. The simulated spectral function in panel e now very closely resembles the experimental spectra, including the clear superconducting gap, the strong spectral weight near E_F, the kink and peak-dip-hump features, etc., all of which originate from the form of the self-energy.

In the final step (Supplementary Fig. 2g), we convolve the spectrum with an energy and momentum broadening function. The energy broadening is the measured energy resolution that is calibrated from the gold Fermi edge taken near the sample position with the same experimental conditions as the data from the superconductor. For this spectrum taken with 24 eV photons, the total energy resolution is 10.5 meV. The momentum broadening is added in to account for various intrinsic and extrinsic broadening factors, such as the angular-resolution of the electron analyzer, the imperfections of the cleaved surface (like surface warpage), and the doping heterogeneity of the single crystal sample that has been commonly observed in scanning tunneling spectroscopy[2]. This extrinsic momentum broadening can vary slightly with different samples, cleaved surfaces or even different photoemission spots on the same cleaved surface. Thus, we put in the momentum broadening as one of the fitting parameter for the low temperature spectrum and then hold this constant for all fits as a function of temperature. For the

spectrum shown in Supplementary Fig. 2g, the momentum broadening is 25 mÅ^{-1}. In Supplementary Fig. 2h, we compare the two different MDC cuts from the spectrum with (red) and without (blue) additional momentum broadening.

Supplementary Note 4. Terms in the self-energy

As discussed in Supplementary Note 2, 8 terms are utilized to parameterize the self-energy for each slice, and two terms ($\mathbf{k}_F$ and the band bottom) are used to parameterize the bare band structure. Along with the energy and momentum broadening terms, the superconducting gap, and the prefactor for the spectral function, the experimental spectra can be very well fit.

In our study, we used the 8 terms to parameterize $\Sigma''(\omega)$ for each experimental cut, letting the energy dependence of $\Sigma'(\omega)$ to be fully determined by the Kramers-Kronig relations. We found that as long as the 8 terms give enough freedom to the fits, the exact functional form of $\Sigma''(\omega)$ is not so important, indicating the overall robustness of the fitting procedure.

Two forms of Σ'' that we utilized are:

$$\Sigma''(\omega) = \lambda\sqrt{\omega^2 + (\pi k_\mathrm{B}T)^2} + \frac{I_1}{e^{\frac{\omega - E_1}{W_1}} + 1} + I_2 e^{\frac{-(\omega - E_2)^2}{2W_2^2}} + \Gamma_0 \qquad (5)$$

$$\Sigma''(\omega) = (\lambda\sqrt{\omega^2 + (\pi k_\mathrm{B}T)^2} + \Gamma_0) \times (\frac{I_1}{e^{\frac{\omega - E_1}{W_1}} + 1} + 1 - I_1 + I_2 e^{\frac{-(\omega - E_2)^2}{2W_2^2}}). \qquad (6)$$

Each of these is essentially a Marginal Fermi Liquid together with a step function at E_1 and a Gaussian function peak at E_2. All of the fits shown in the main paper were obtained using the form of Supplementary Eq. (5), while the Supplementary Fig. 3 shows a comparison of results obtained using the two functional forms.

Supplementary Eq. (5) and (6) contain the 8 parameters λ, Γ_0, I_1, E_1, W_1, I_2, E_2, and W_2. An extensive study of the temperature and energy dependence of the scattering rates in the normal state of the cuprates indicated that λ for nodal states of optimally doped samples should be near 0.5 [8]. We found that this value worked well for the temperature dependence set of data shown in Fig. 1, thus we set it to 0.5 for the momentum dependent set of data at 15 K shown in Fig. 2.

An additional term we utilized is a cutoff energy ω_c that brings $\Sigma''(\omega)$ to zero at high frequencies, which is necessary for the Kramers-Kronig transformation to work. Kordyuk *et al.* [9] have reported the cutoff energy scale to be around 500meV - our finding of the cutoff energy is consistent with that result.

Supplementary Note 5. Energy-dependent complex order parameter and superconducting gap

In conventional strongly coupled superconductors, it is found that the pairing interaction can be strongly retarded, which brings in an energy dependence to the pairing order parameter (related to the superconducting gap Δ). Because of causality (the Kramer's-Kronig relations), any energy dependence of the order parameter necessitates that it be complex, having both real and imaginary parts.

To explore this physics, we performed fits with an energy-dependent complex superconducting order parameter. In this case we write $\phi(\omega) = \phi'(\omega) + i\phi''(\omega)$, which then corresponds to an energy-dependent complex superconducting gap $\Delta(\omega) = \phi(\omega)/(1 - \frac{\Sigma(\omega)}{\omega})$. We utilized 5 terms to allow for the energy dependence of $\phi''(\omega)$ as:

$$\phi'' = \frac{I}{e^{\frac{\omega - E_3}{W_3}} + 1} + \frac{I}{e^{\frac{-\omega + E_4}{W_4}} + 1} \tag{7}$$

and utilized the Kramer's-Kronig transformation to obtain $\phi'(\omega)$. As is typical, we also enforced ϕ'' to be odd in ω and ϕ' to be even.

Supplementary Fig. 4 shows a comparison between the fits obtained with the complex energy-dependent ϕ (complex energy-dependent Δ) and the one with a real-only ϕ, where $\phi=Z(\omega)\times\Delta$, and Δ is a real constant. The results for ϕ' and ϕ'' are shown in panel e. Even though there are many more parameters, the results and qualities of the fits with the real-only and the complex ϕ are almost identical. These results clearly show that the energy dependence of ϕ and Δ is much less important than the energy dependence of Σ, i.e. any energy dependence of the pairing interaction is a second-order effect.

Supplementary Note 6. Comparison of 1D and 2D fit results

Supplementary Fig. 5 shows the comparison of the data and the fit in 1D cuts (EDCs and MDCs) from the temperature dependent set of data that is shown in Fig. 1 in the main paper. Three different temperatures of data and fit that lie in different regions of the phase diagram are shown as typical examples. The EDCs and MDCs of the data and fit indicate a very high fitting quality of our 2D fitting method that is comparable to those using phenomenological model fitting to only EDCs or MDCs[10,11,12,13,14,15,16,17], though the fitting using the 2D method effectively contains 100 times fewer fitting parameters.

Supplementary Fig. 6 shows a quasiparticle peak view from $\mathbf{k}_\mathbf{F}$ EDCs. As a complimentary tool to the false color scale spectra, the $\mathbf{k}_\mathbf{F}$ EDCs from spectra of various temperatures in Fig. 1 show the evolution of quasiparticle coherence. The quasiparticle peaks sharply emerge between T_{pair} and T_{C} – behavior that has been qualitatively described in a previous study[16] and attributed to the onset of the superfluid density (a property of the 2-particle pairs) in the superconducting state.

Here, we understand that this is due to the strong evolution of the single particle self-energies $\Sigma'(\omega)$ and $\Sigma''(\omega)$.

Supplementary Note 7. Energy dependent superconducting gap in previous ARPES studies

A previous ARPES study[15] reported a significant energy dependence to the complex superconducting gap and a complex order parameter. This contrasts with our results (Supplementary Fig. 4), which are equally well fit with a simpler real static gap. Here we discuss a few other differences between the two studies:

- The previous study was limited to the near-node and mid-zone regions, where the kink strengths are much weaker.
- The previous study exclusively utilized the conventional 1D MDC fitting method, since the 2D method was not available to them at that time. This includes momentum cuts inside the gap region which do not have any true poles and where there are many fewer constraints. The self-energies extracted in this gap region showed a very strong or even dominant peak that they attribute to impurities[15,18]. However, unlike the ~55 meV kink feature, this strong low-energy feature in the self-energy cannot be observed directly from the corresponding ARPES spectrum. Such an extra peak is not present in the self-energies that we extract with the 2D fitting method.

Our results show that the single particle self-energy and its energy dependence are much more important than any effects of the energy dependence of the superconducting gap or order parameter, and that these self-energy effects are in general critical to the physics of the cuprates.

Supplementary Note 8. Kinks and electron-boson coupling from previous ARPES studies

Previous ARPES studies of cuprates have discovered dispersion "kinks" or mass enhancements near the Fermi level in the near-nodal[19,20,21] and antinodal regime[11,12,13] which have been generally interpreted as indicating electron-boson coupling, with the debate centering on whether the boson is phononic or magnetic in origin, or both[22,23,24]. It is important to distinguish the near-nodal and antinodal regimes, as these behave quite differently - the near-nodal kinks are generally significantly weaker, are at slightly higher energy, and have only a weak temperature dependence. The case for electron-boson coupling, and electron-phonon coupling in particular, for these near-nodal states is relatively strong, including an isotope effect study on the near-nodal kinks[21]. In contrast, the mid-zone and antinodal kinks discussed in the present work are much stronger than these nodal kinks, with a renormalization factor (6.5 at the antinode, as shown in Fig. 3c) that is so strong we argue that it is beyond what is possible from perturbative physics such as is at the heart of the Eliashberg equations that are typically used for the description of electron-boson coupling in conventional strongly-coupled superconductors.

Supplementary Note 9. Particle-hole symmetry

To justify our assumption of particle-hole symmetry on the self-energy ($\Sigma''(\omega)$ to be even and $\Sigma'(\omega)$ to be odd over E_F), in Supplementary Fig. 7, we show a set of 2D fitting results with the self-energy that is enforced to be particle-hole asymmetric. The intensity on the hole side of the $\Sigma''(\omega)$ is forced to be larger or smaller than the particle side (Supplementary Fig. 7a). The goodness of the fits – the chi-squared values shown in Supplementary Fig. 7c indicate that the fit with particle-hole symmetry has the best fit quality (the smallest chi-square value). On the other hand, Supplementary Figs. 7a-b show that even under different conditions of particle-hole asymmetry in the self-energy, both $\Sigma''(\omega)$ and $\Sigma'(\omega)$ (inset in Supplementary Fig. 7b) reveal an

energy-dependent behavior that is quantitatively robust on the particle (occupied) side, except for a constant shift in $\Sigma'(\omega)$. The extracted spectral gap sizes in Supplementary Fig. 7d also show a small variation (the standard deviation is only 0.7meV for the average 20meV gap size). In Supplementary Fig. 7e-k, we show the corresponding fit results of different particle-hole symmetric and asymmetric conditions together with the bare band dispersion. The extracted bare band dispersion (Supplementary Fig. 7l) shows a shift in energy, which compensates the shift in $\Sigma'(\omega)$.

As ARPES only probes the particle (occupied) side of the spectral function, the particle-hole symmetry has long been an assumption in the analysis of ARPES spectra, especially in the commonly used symmetrized EDC method[10] that proved to be a useful technique to extract the spectral gap size and the scattering rate. In the symmetrized EDC method, both the density of states and self-energy is assumed to be symmetric, whereas our 2D fitting technique only assumes the symmetry on self-energy but not on the density of states. Based on the discussion above, we can conclude that the particle-hole asymmetry on self-energy only brings in minor effects to our results, where the behavior of both $\Sigma'(\omega)$ and $\Sigma''(\omega)$ is quantitatively robust. And the particle-hole symmetry assumption is justified as judged by the minimization of the chi-square value.

Our result shows the assumption of particle-hole symmetry gives the best goodness of fit. We note that DMFT calculations have predicted some particle-hole asymmetry appearing in the self-energy [25]. To our knowledge, our result is the first experimental data able to address this question, with our finding in opposition to this prediction.

Supplementary Note 10. Momentum range of pairing interactions

The effective momentum range of particle-hole mixing shown in Fig. 3 is the full width at half maximum (FWHM) of the particle-hole mixing probability $u_{\mathbf{k}}^2 v_{\mathbf{k}}^2 = \frac{\Delta^2}{4(\varepsilon_{\mathbf{k}}^2+\Delta^2)}$, where $v_{\mathbf{k}}^2 = \frac{1}{2}(1 - \frac{\varepsilon_{\mathbf{k}}^2}{\varepsilon_{\mathbf{k}}^2+\Delta^2})$, and $u_{\mathbf{k}}^2 = \frac{1}{2}(1 + \frac{\varepsilon_{\mathbf{k}}^2}{\varepsilon_{\mathbf{k}}^2+\Delta^2})$ are the coherence factors for particles and holes respectively, in which $\varepsilon_{\mathbf{k}}$ is the ungapped electron dispersion, and Δ is the superconducting gap. The value of $u_{\mathbf{k}}^2 v_{\mathbf{k}}^2$ indicates the probability of particle-hole mixing. As $u_{\mathbf{k}}^2 v_{\mathbf{k}}^2$ peaks at $\mathbf{k}_F$, where $\varepsilon_{\mathbf{k}} = 0$, the FWHM of $u_{\mathbf{k}}^2 v_{\mathbf{k}}^2$ is where $\varepsilon_{\mathbf{k}} = \pm\Delta$, thus the electron energy at the half maximum is $E_{\mathbf{k}} = \sqrt{\varepsilon_{\mathbf{k}}^2 + \Delta^2} = \sqrt{2}\Delta$. Considering the renormalization effect, $\varepsilon_{\mathbf{k}}$ is then equal to $\xi_{\mathbf{k}}/Z$, where $\xi_{\mathbf{k}}$ is the bare electron band dispersion and Z is the renormalization factor discussed in the paper and in Supplementary Note 11 below. The particle-hole mixing range of the full Brillouin zone shown in Fig. 3d is calculated with the $\xi_{\mathbf{k}}$, Z and Δ extracted from the 2D fitting of the ARPES spectra shown in Fig. 2, after interpolating the parameters of those five cuts to the whole Brillouin zone.

Supplementary Note 11. Renormalization factor and quasiparticle residue

The renormalization factor and quasiparticle residue are both commonly denoted by Z, even though they are actually inverses of each other. In this paper, we call the renormalization factor $Z = 1 - \frac{\Sigma'}{\omega}$, or, for the limit of $\omega \to 0$, $Z = 1 - \frac{\partial \Sigma'}{\partial \omega}\Big|_{\omega \to 0}$. The renormalization factor represents the strength of the band renormalization effect at the $\mathbf{k}_F$, which is consistent with the mass enhancement value in a metal. In this terminology, the quasiparticle residue would have weight $1/Z$.

Supplementary Note 12. Pseudogap

In underdoped cuprates, a commonly known feature "pseudogap" that can be observed at much higher temperature scale well above the T_{pair} has attracted a lot of interest in high-T_{C} superconductivity research[26]. Though the pseudogap is presented in the (underdoped) sample we studied, its presence is a relatively minor effect, which is why we don't discuss it prominently in the paper. Supplementary Fig. 8 shows a comparison of spectra taken at the mid-zone and near the antinode with temperature below and above T_{C}. The symmetrized $\mathbf{k_F}$ EDC of the high temperature (150 K) spectrum taken near the antinode displays a spectral weight depletion with weak coherence peak near the Fermi level (pseudogap) that is absent in the mid-zone. This k-dependence is consistent with previous reports[27]. This pseudogap feature is observable up to a temperature scale T^* that is much higher than the pairing temperature scale T_{pair} mentioned in the present paper. This difference in these two temperature scales is consistent with other recent findings[28,29,30]. On the other hand, both the mid-zone and the near antinodal spectra exhibit a similar trend when moving into the superconducting state. At high temperature (150 K), they both have broad and incoherent spectra, whereas at temperatures well below T_{C} (50 K), the spectra display sharp and strongly renormalized quasiparticle peaks. This is also revealed by the strong quasiparticle peaks in the symmetrized $\mathbf{k_F}$ EDCs at low temperature compared to the broad curves at high temperature. The change in the general behavior is observed to begin at T_{pair} (Fig. 3a), i.e. it is dominated by the pairing physics rather than the pseudogap physics.

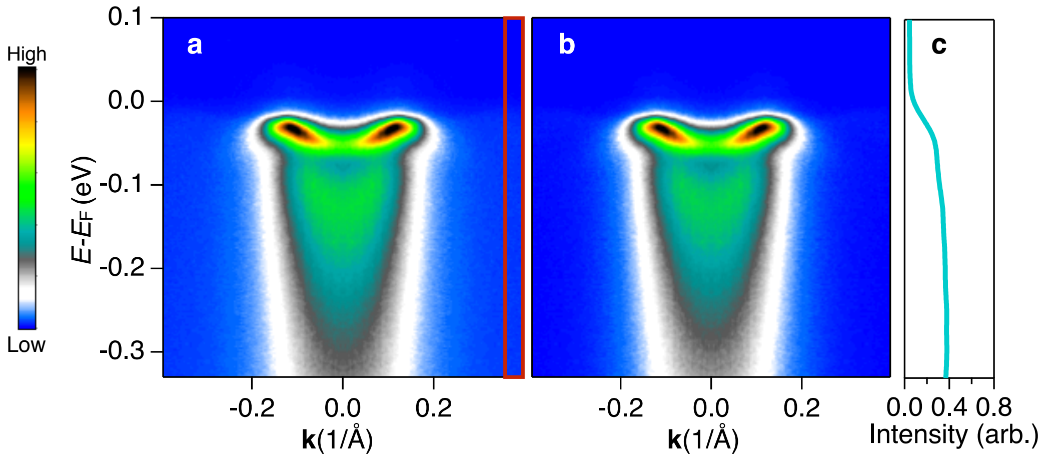

Supplementary Figure 1 | Energy dependent background subtraction. Example spectrum without (panel **a)** and with (panel **b**) background subtraction. (**c**) The energy-dependent background, which is the averaged and smoothed EDC within the red box in panel **a**.

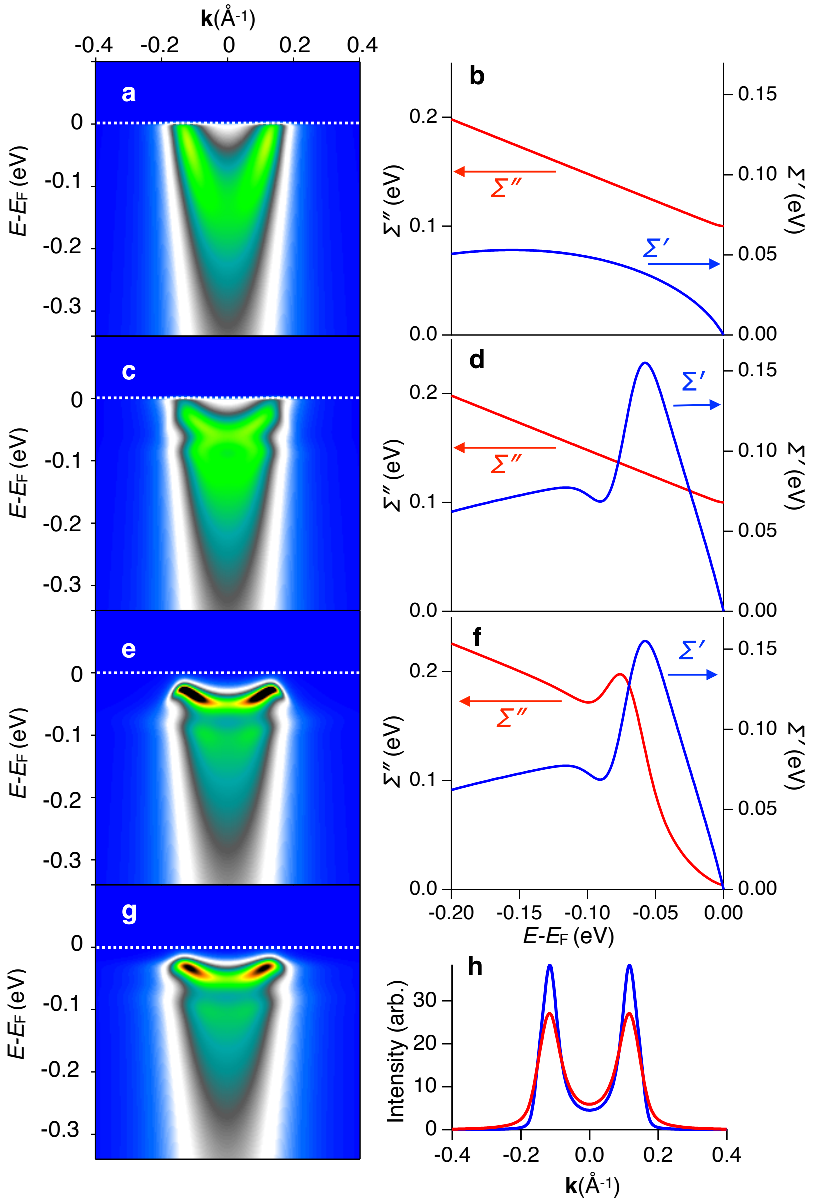

Supplementary Figure 2 | Phenomenological construction of the self-energy. Simulated spectra illustrating the effect of various forms of self-energy (see Supplementary Note 3 for details). The self-energies of panel **d** are not causally self-consistent, and are shown for illustrative purposes only. Color scales are the same for spectrum in panel **a** to **g**. **h,** The comparison of MDCs with (red) and without (blue) extrinsic broadening.

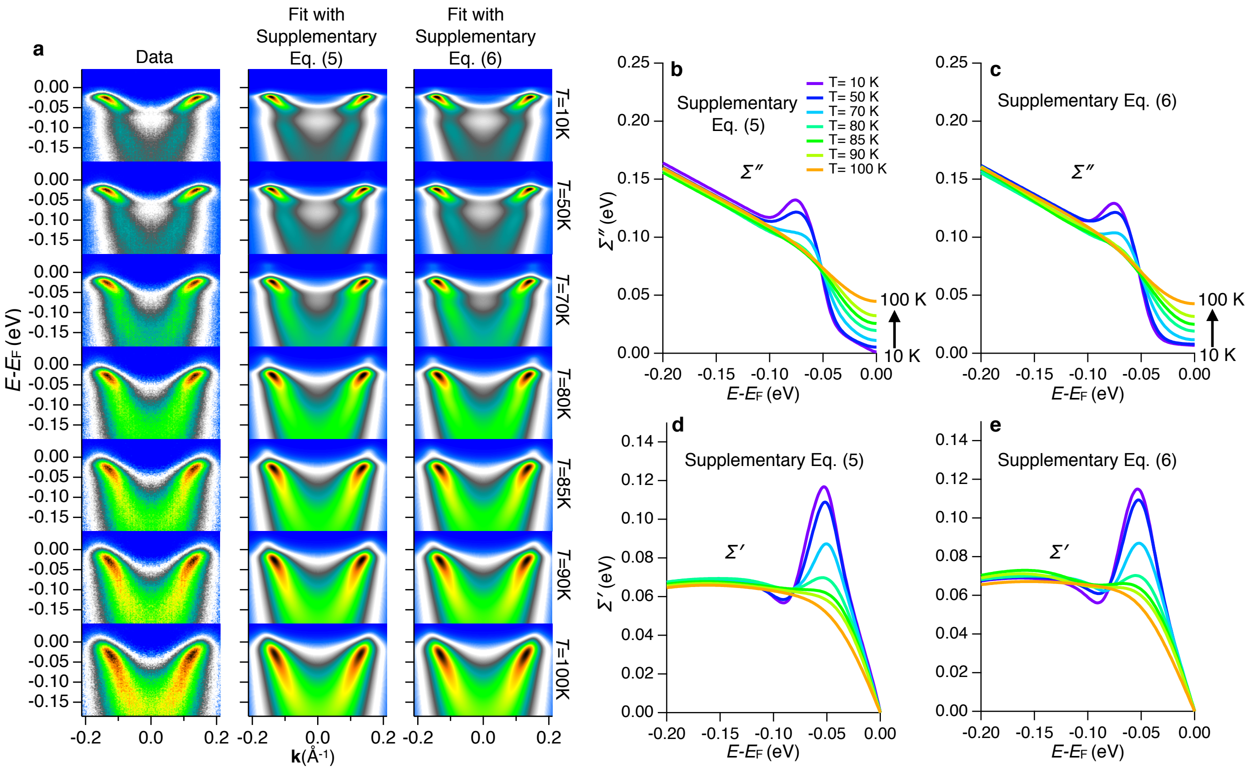

Supplementary Figure 3 | Self-energy constructed with different functional forms. (**a**) Data and the fits with two different functional form (see Supplementary Note 4 for details) to construct the imaginary self-energy. (**b, c**) The imaginary part of the self-energy extracted from the fittings. (**d, e**) The real part of the self-energy that extracted from the fitting.

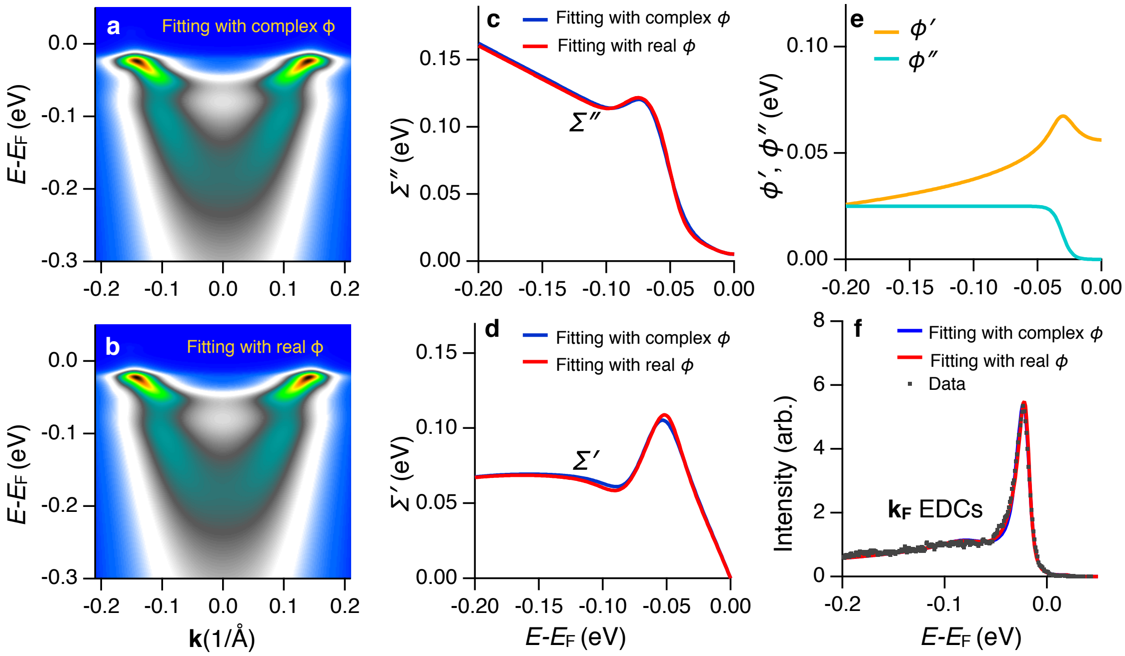

Supplementary Figure 4 | Fitting with a complex superconducting order parameter. (**a**) Fitting the spectrum of Fig. 1a in the main text with a complex superconducting order parameter ϕ (see Supplementary Note 5 for details). (**b**) The fit result with only a real ϕ (what we used for all other fitting in the paper), the color scale is the same with panel **a**. (**c, d**) Imaginary and real part of self-energy for these two different fittings. (**e**) The complex order parameter for the spectrum in panel **a**. (**f**) The $\mathbf{k}_F$ EDC of spectra in panel **a** and **b** and the experiment data that they fit to.

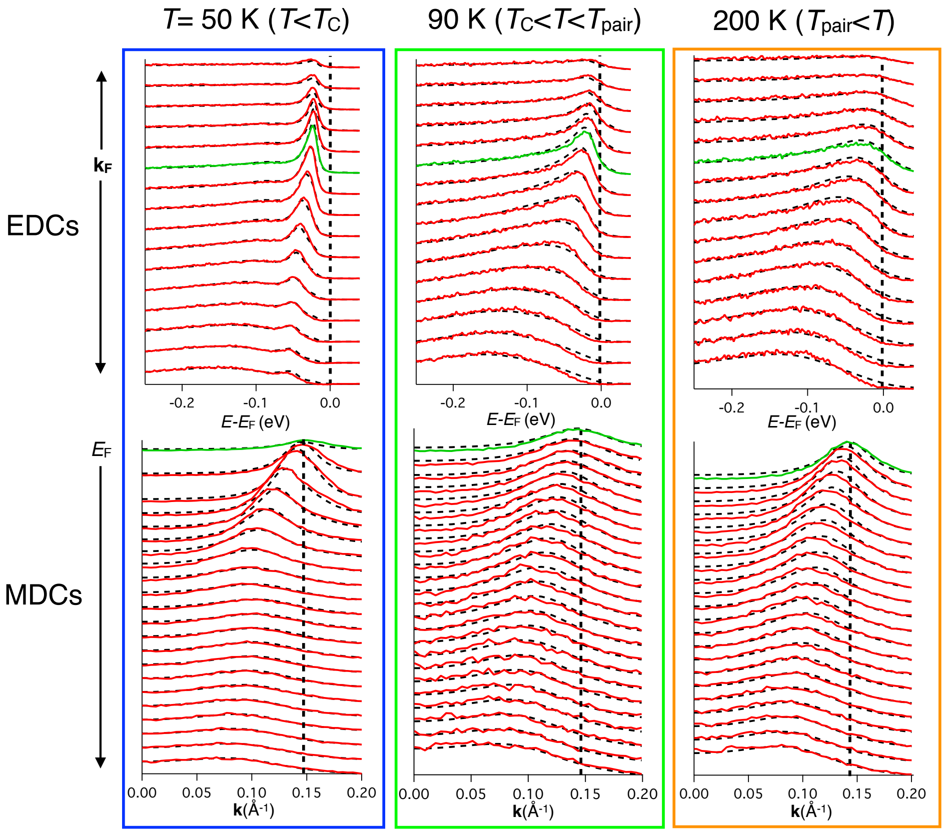

Supplementary Figure 5 | MDC and EDC line cuts from the experimental data (red solid) and fits (black dashed) from the mid-zone θ=22.5° color scale plot of Fig. 1 and Supplementary Fig. 3. In contrast to the standard method in which all EDCs and MDCs are individually fit and individually scaled in amplitude, all spectra for one temperature were fit simultaneously, with no additional amplitude scaling or other modifications. This is a much more severe constraint than utilized in previous fitting studies of ARPES data.

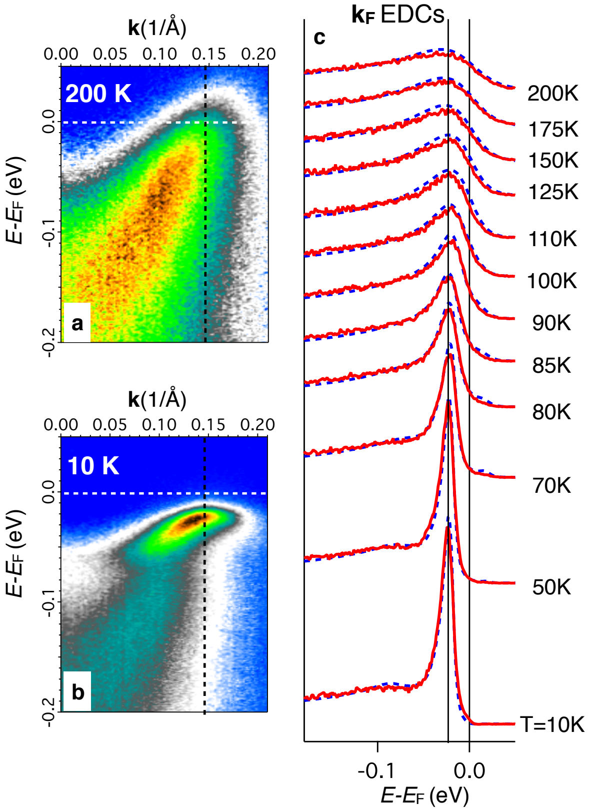

Supplementary Figure 6 | Temperature dependence of the quasiparticle peaks, from the mid-zone θ=22.5° cut. (**a, b**) 2D ARPES spectra at two extreme temperatures. (**c**) EDCs (red solid) from these and similar plots at $\mathbf{k}=\mathbf{k}_F$, i.e. along the vertical black dashed line in panel **a** and **b**. Line cut through the 2D fits to these data are shown as the blue dashed lines in panel **c**. The quasiparticle peak sharply emerges between T_{pair} and T_{C}.

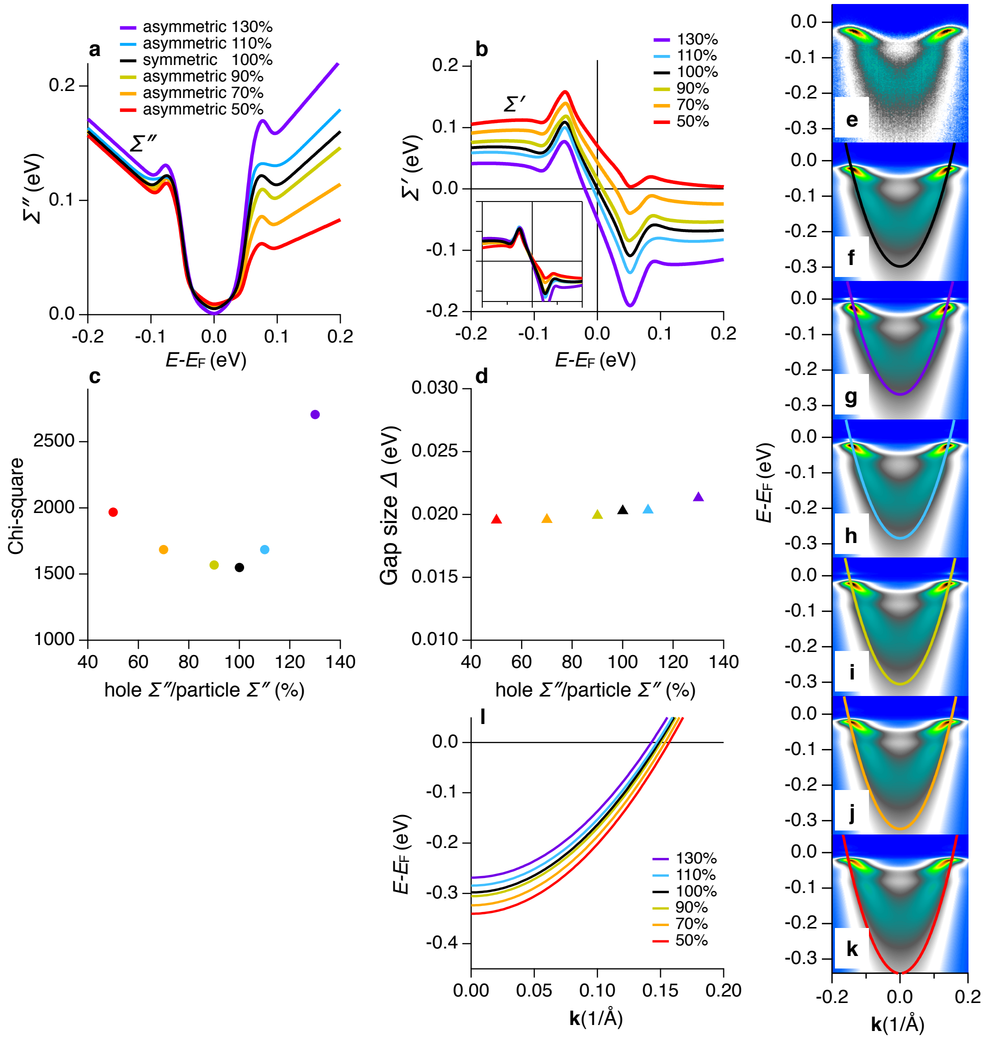

Supplementary Figure 7 | 2D fitting with enforcing particle-hole asymmetry. (**a**) The extracted imaginary part of the self-energy $\Sigma''(\omega)$ from 2D fitting, with the intensity of $\Sigma''(\omega)$ on the hole side enforced to be 130%, 110%, 100% (particle-hole symmetric), 90%, 70%, and 50% of the particle (occupied) side. (**b**) The correspondent real part of the self-energies $\Sigma'(\omega)$, where the inset shows the $\Sigma'(\omega)$ with the offset to be $\Sigma'(0) = 0$. (**c**) The statistical chi-square value of each fit, indicating the goodness of the fit (the smaller the chi-square value, the better the fit). (**d**) The spectral gap size from each 2D fitting, which shows a small variation with standard deviation to be 0.7 meV comparing with the average gap size of 20 meV. (**e**) ARPES spectrum of the mid-zone cut taken at 50 K (same as in Fig. 1a). (**f-k**) The fit spectra with different particle-hole symmetry and asymmetric condition, where **f** is the same fit as in Fig. 1a with the assumption of particle-hole symmetry, **g** to **k** are the spectra with particle hole asymmetry where the intensity of $\Sigma''(\omega)$ at the hole side is 130%, 110%, 90%, 70%, 50% of the particle side respectively. The colored curves are the correspondent bare band dispersions. (**l**) The extracted bare band dispersions.

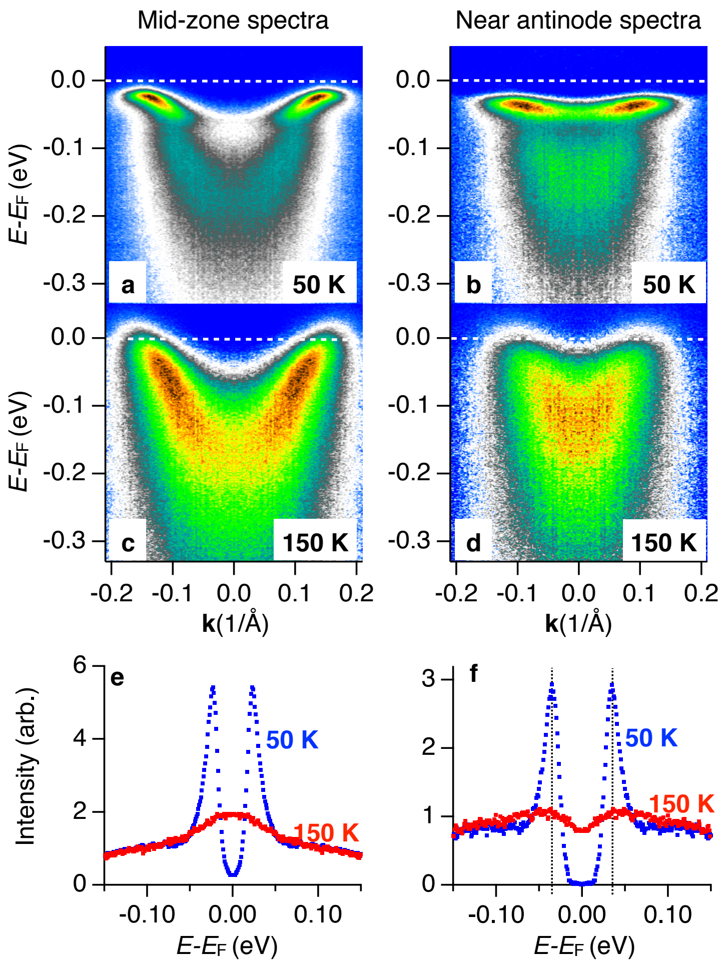

Supplementary Figure 8 | Comparing spectra at mid-zone and near antinodal region. (**a**, **b**) Low temperature spectra (T=50 K) taken at the mid-zone (Fermi surface angle at 22.5 degree) and near the antinode (32 degree). (**c**, **d**) The same set of spectra taken at 150 K. (**e, f**) Symmetrized $\mathbf{k_F}$ EDCs of the mid-zone and near antinodal spectra at 50 K and 150 K. The correspondent symmetrized EDC of the near antinodal spectrum at 150 K shows a strong depletion of the spectral weight near Fermi level (pseudogap), whereas the mid-zone one doesn't show any gap feature at 150 K.

Supplementary References:

1. Norman, M. R., Eschrig, M., Kaminski, A. & Campuzano, J. C. Momentum distribution curves in the superconducting state. *Phys. Rev. B* **64**, 184508 (2001).

2. Alldredge, J. W. *et al.* Evolution of the electronic excitation spectrum with strongly diminishing hole density in superconducting. *Nat. Phys.* **4**, 319-326 (2008).

3. Gorkov, L. P. On the energy spectrum of superconductors. *J. Exp. Theoret. Phys. (U.S.S.R)* **34**, 735-739 (1958).

4. Nambu, Y. Quasi-particles and gauge invariance in the theory of superconductivity. *Phys. Rev.* **117**, 648 (1960).

5. Dahm, T. *et al.* Strength of the spin-fluctuation-mediated pairing interaction in a high-temperature superconductor *Nat. Phys.* **5**, 217-221 (2009).

6. Inosov, D. S. *et al.* Relation between the one-particle spectral function and dynamic spin susceptibility of superconducting $Bi_2Sr_2CaCu_2O_{8+\delta}$. *Phys. Rev. B* **75**, 172505 (2007).

7. Varma, C. M., Littlewood, P. B., Schmitt-Rink, S., Abrahams, E., & Ruckenstein, A. E. Phenomenology of the normal state of Cu-O high-temperature superconductors. *Phys. Rev. Lett.* **63**, 1996 (1989).

8. Reber, T. J. *et al.* Power law liquid - a unified form of low-energy nodal electronic interactions in hole doped cuprate superconductors. Preprint at https://arxiv.org/abs/1509.01611 (2015).

9. Kordyuk, A. A. *et al.* Bare electron dispersion from experiment: Self-consistent self-energy analysis of photoemission data. *Phys. Rev. B* **71**, 214513 (2005).

10. Norman, M. R., Randeria, M., Ding, H. & Campuzano, J. C. Phenomenology of the low-energy spectral function in high-*T*c superconductors. *Phys. Rev. B* **57**, R11093(R) (1998).

11. Kaminski, A. *et al.* Renormalization of spectral line shape and dispersion below T_C in $Bi_2Sr_2CaCu_2O_{8+\delta}$. *Phys. Rev. Lett* **86**, 1070 (2001).

12. Kim, T. K. *et al.* Doping dependence of the mass enhancement in $(Pb,Bi)_2Sr_2CaCu_2O_8$ at the antinodal point in the superconducting and normal states. *Phys. Rev. Lett.* **91**, 167002 (2003).

13. Gromko, A. D. *et al.* Mass-renormalized electronic excitations at (π, 0) in the superconducting state of $Bi_2Sr_2CaCu_2O_{8+\delta}$. *Phys. Rev. B* **68**, 174520 (2003).

14 . Cuk, T. *et al.* Coupling of the B_{1g} phonon to the antinodal electronic states of $Bi_2Sr_2Ca_{0.92}Y_{0.08}Cu_2O_{8+\delta}$. *Phys. Rev. Lett.* **93**, 117003 (2004)

15. Bok, J. M. *et al.* Quantitative determination of pairing interactions for high-temperature superconductivity in cuprates. *Sci. Adv.* **2**, 1501329 (2016).

16. Feng, D. L. *et al.* Signature of superfluid density in the single-particle excitation spectrum of $Bi_2Sr_2CaCu_2O_{8+\delta}$. *Science* **289**, 277-281 (2000).

17. Bok, J. M. *et al.* Momentum dependence of the single-particle self-energy and fluctuation spectrum of slightly underdoped $Bi_2Sr_2CaCu_2O_{8+\delta}$ from high-resolution laser angle-resolved photoemission. *Phys. Rev. B* **81**, 174516 (2010).

18. Hong, S. H. *et al.* Sharp low-energy feature in single-particle spectra due to forward scattering in d-wave cuprate superconductors. *Phys. Rev. Lett.* **113**, 057001 (2014).

19. Lanzara, A. *et al.* Evidence for ubiquitous strong electron–phonon coupling in high-temperature superconductors. *Nature* **412**, 510-514 (2001).

20. Johnson, P. D. *et al.* Doping and temperature dependence of the mass enhancement observed in the cuprate $Bi_2Sr_2CaCu_2O_{8+\delta}$. *Phys. Rev. Lett.* **87**, 177007 (2001).

21. Iwasawa, H. *et al.* Isotopic fingerprint of electron-phonon coupling in high-T_C Cuprates. *Phys. Rev. Lett* **101**, 157005 (2008).

22. Damascelli, A., Hussain, Z. & Shen, Z.-X. Angle-resolved photoemission studies of the cuprate superconductors. *Rev. Mod. Phys.* **75**, 473 (2003).

23. Cuk, T. *et al.* A review of electron–phonon coupling seen in the high-*T*c superconductors by angle-resolved photoemission studies (ARPES). *Phys. Stat. Sol.* (b) **242**, No. 1, 11–29 (2005).

24. Kordyuk, A. A. *et al.* Constituents of the quasiparticle spectrum along the nodal direction of high-T_C cuprates. *Phys. Rev. Lett.* **97**, 017002 (2006).

25. Berthod, C. *et al.* Non-Drude universal scaling laws for the optical response of local Fermi liquids. Phys. Rev. B **87**, 115109 (2013).

26. Hashimoto, M., Vishik, I. M., He, R.-H., Devereaux, T. P. & Shen, Z.-X. Energy gaps in high-transition-temperature cuprate superconductors. *Nat. Phys.* **10**, 483–495 (2014).

27. Tanaka, K. *et al.* Distinct fermi-momentum-dependent energy gaps in deeply underdoped Bi2212. *Science* **314**, 1910–1913 (2006)

28. Kondo, T. *et al.* Disentangling Cooper-pair formation above the transition temperature from the pseudogap state in the cuprates. *Nat. Phys.* **7**, 21–25 (2011).

29. Reber, T. J. *et al.* Prepairing and the "filling" gap in the cuprates from the tomographic density of states. *Phys. Rev. B* **87**, 060506(R).

30. Wang, Y. *et al.* Onset of the vortexlike Nernst signal above T_C in $La_{2-x}Sr_xCuO_4$ and $Bi_2Sr_{2-y}La_yCuO_6$. *Phys. Rev. B* **64***,* 224519 (2001).
